# Ketogenic Diet in the Treatment of Epilepsy

**DOI:** 10.3390/nu16091258

**Published:** 2024-04-24

**Authors:** Kinga Borowicz-Reutt, Marlena Krawczyk, Julia Czernia

**Affiliations:** Independent Unit of Experimental Neuropathophysiology, Department of Toxicology, Medical University of Lublin, Jaczewskiego 8b, PL-20-090 Lublin, Poland; marlena.krawczyk1@umlub.pl (M.K.); julia.czernia@umlub.pl (J.C.)

**Keywords:** ketogenic diet, KD, seizure, epilepsy, efficacy, safety

## Abstract

Epilepsy is one of the most disabling neurological diseases. Despite proper pharmacotherapy and the availability of 2nd and 3rd generation antiepileptic drugs, deep brain stimulation, and surgery, up to 30–40% of epilepsy patients remain drug-resistant. Consequences of this phenomenon include not only decreased a quality of life, and cognitive, behavioral, and personal disorders, but also an increased risk of death, i.e., in the mechanism of sudden unexpected death in epilepsy patients (SUDEP). The main goals of epilepsy treatment include three basic issues: achieving the best possible seizure control, avoiding the undesired effects of treatment, and maintaining/improving the quality of patients’ lives. Therefore, numerous attempts are made to offer alternative treatments for drug-resistant seizures, an example of which is the ketogenic diet. It is a long-known but rarely used dietary therapy for intractable seizures. One of the reasons for this is the unpalatability of the classic ketogenic diet, which reduces patient compliance and adherence rates. However, its antiseizure effects are often considered to be worth the effort. Until recently, the diet was considered the last-resort treatment. Currently, it is believed that a ketogenic diet should be used much earlier in patients with well-defined indications. In correctly qualified patients, seizure activity may be reduced by over 90% or even abolished for long periods after the diet is stopped. A ketogenic diet can be used in all age groups, although most of the available literature addresses pediatric epilepsy. In this article, we focus on the mechanisms of action, effectiveness, and adverse effects of different variants of the ketogenic diet, including its classic version, a medium-chain triglyceride diet, a modified Atkins diet, and a low glycemic index treatment.

## 1. Introduction

Despite the availability of three generations of antiepileptic drugs and their remarkable effectiveness, these medications do not provide complete control in 30–40% of epilepsy patients. Therapy with old generation antiseizure medications may lead to adverse effects in the central nervous system (sedation, fatigue, dizziness, coordination disorders, tremors, cognitive deficits, mood alterations, and behavioral changes), hepatotoxicity, and teratogenicity, and the last two are mostly attributed to valproate. Additionally, these drugs easily interact with other medications. Newer antiseizure drugs did not prove to be superior in terms of effectiveness. However, some of them may be better tolerated and have simpler pharmacokinetics, thereby having lower interaction potential. New generation antiepileptic drugs were designed to have fewer side effects compared to older ones. In fact, the most commonly described central nervous system-related adverse effects appear less frequently. Nevertheless, the 2nd and 3rd generations of antiepileptic drugs did not improve response rates in patients with intractable epilepsy. Therefore, alternative treatments are being introduced, an example of which is the ketogenic diet (KD) [1].

The history of ketogenic diet therapy dates back to the times of Hippocrates, when seizures were treated via dietary restrictions. The first mention of the beneficial effects of fasting in epilepsy can be found in the New Testament. In more modern times, around 150 years ago, a boy with seizures was successfully treated with starvation by a faith healer. As knowledge about metabolism increased, fasting was replaced by a high-fat diet. However, the introduction of phenytoin in 1938, and the further development of other antiepileptic drugs, declined the use of the KD almost completely. In 1994, the diet gained attention again when a two-year-old boy with intractable epilepsy underwent such therapy at Johns Hopkins Hospital. He achieved a seizure-free state and his psychophysical development accelerated [2,3].

The KD is an extremely low-carb, high-fat, and adequate or low-protein diet (tailored to the individual’s requirements) inducing the production of ketone bodies. Clinical studies showed that this diet can be an effective treatment for drug-resistant epilepsy primarily in children and adolescents [4]. Due to the high attrition rates, alternative and more palatable variants of KDs were designed to reduce adverse effects and improve patient compliance and satisfaction. Presently, the following variants of the KD are available:

a. The classic ketogenic diet (cKD)
b. A medium-chain triglyceride (MCT) diet
c. A modified Atkins diet (MAD)
d. A low glycemic index treatment (LGIT)

In general, KDs can be used in all age groups. However, most data from the literature confirms the good tolerance and high effectiveness of this diet in infants and children (up to 10 years of age), in whom seizure freedom is often achieved and maintained. Therefore, the most detailed recommendations for the use of the KD refer to patients of this age (see Table 1). Despite previous concerns regarding the immaturity of the liver and lipid-metabolizing enzymes, KDs were utilized effectively and safely even for infants as young as 6 weeks.

Infants enter into ketosis more easily and respond better to the diet treatment. Early introduced, aggressive, and optimal therapy with the KD can have a positive impact on the neurodevelopment of children with infantile spasms (West syndrome), Ohtahara syndrome, an epilepsy of infancy with migrating seizures, and a resistant epilepsy with focal seizures awaiting epilepsy surgery. The indications for the early use of the KD include conditions in which at least three publications (from at least two ketogenic diet treatment centers) reported at least a 20% increase in antiseizure action above the average for the KD. Since this average is defined as a 40–50% chance of ≥50% seizure reduction, early diet treatment is indicated in epilepsy syndromes with 60–70% responder rates. Conditions in which around 50% of patients achieve ≥50% of seizure frequency reduction are referred to as disorders that respond modestly to the KD. According to recommendations, this diet is particularly indicated in casuistic cases of glucose transporter protein 1 glucose transporter (GLUT1) deficiency, in which glucose cannot enter the brain cells, and in pyruvate dehydrogenase deficiency, where ketone bodies can omit the enzymatic blockade. In both disorders, the KD provides ketones that serve as an alternative cerebral fuel for the developing brain. There are age-specific recommendations for the use of KD in GLUT-1 deficiency syndrome. In infants and preschool children, the classic KD should be used at first and maintained as long as possible. An MAD is an effective alternative in school children, adolescents, and adults. Traditionally, the KD has been recommended as a last treatment option in patients with intractable seizure disorders; however, the beneficial effects of diet therapy justify its earlier use. Drug-resistant epilepsy is defined as the failure of adequate trials of two tolerated, appropriately chosen and used antiepileptic drug schedules, either in monotherapies or drug combinations, to achieve sustained seizure freedom. According to the International Ketogenic Diet Study Group, the KD should be introduced when an average of 2.6 ± 0.9 antiepileptic drugs have proven to be ineffective. The most common examples of refractory epilepsy are Lennox–Gastaut syndrome, West syndrome, myoclonic-astatic epilepsy, Dravet syndrome, tuberous sclerosis, Landau–Kleffner syndrome, and Rett syndrome. The KD is also effective in treating generalized epilepsy, including tonic-clonic, myoclonic, atonic, and myoclonic-astatic seizures. Much less efficacy was noticed in complex partial seizures; however, patients with partial-onset seizures can greatly benefit from this dietary regime. Also, in people whose seizures are considered to be uncontrollable with very limited or no further therapeutic options, KDs can be considered. Theoretically, KDs may be suggested to patients for whom the available alternative treatments appear to be less effective and/or induce more serious adverse effects. A multicenter study showed no relationship between KD outcomes and factors like age, sex, seizure type, or baseline EEG recording. Importantly, all variants of KDs are absolutely contraindicated in cases of rare, inherited metabolic errors related to lipid and pyruvate metabolism, an inability to maintain adequate nutrition, a surgical focus identified by neuroimaging and video-EEG monitoring, parent or caregiver noncompliance, and propofol co-treatment increasing the risk of propofol infusion syndrome. Such clinical symptoms as developmental delay, cardiomyopathy, hypotonia, exercise intolerance, myoglobinuria, and easy fatigability indicate that inborn metabolic errors should be excluded before the decision to start a KD. Since implementation challenges, dietary compliance issues, and adverse effects can lower the acceptance rate, candidates for KD treatment should be carefully selected according to the inclusion criteria [3,4,5,6,7,8].

Due to the limited trials in adolescents and adults with epilepsy, the determination of the effectiveness and tolerability of KDs in these age groups was particularly difficult. It is believed that the KD should be offered to adolescents and adults with epilepsy syndromes that developed in childhood and responded well to this treatment. Some examples are tuberous sclerosis complex, Rett syndrome, Lennox–Gastaut syndrome, GLUT1 deficiency syndrome, genetic generalized epilepsies, and focal epilepsies due to underlying migrational disorders. However, adults with drug-resistant focal epilepsy should have surgical treatment proposed to them first, due to the greater likelihood of it achieving a seizure-free state. Some case reports demonstrated the safety and effectiveness of the KD in the treatment of new-onset refractory status epilepticus and its subset febrile infection-related epilepsy syndrome with status epilepticus. Several clinical trials demonstrated the effectiveness of this diet in adult patients with drug-resistant focal, multifocal, and generalized epilepsies [9,10,11,12,13].

The classic KD is a high-fat, adequate or low-protein, and low-carbohydrate diet regime. The lipid-to-nonlipid (total protein and carbohydrate) weight ratio, known as the ketogenic proportion, is usually determined as 4:1, 3:1, or 2:1. This means that for every 4, 3 or 2 g of fat overall, there is 1 g of proteins and carbohydrates. The most desired in clinical conditions is the 4:1 proportion, providing 80% of the total energy from fat, primarily long-chain triglycerides, 15% from proteins, and 5% from carbohydrates. Long-chain triglycerides are then metabolized to shorter particles, i.e., medium-chain fatty acids. For infants and adolescents, who are still developing, the optimum proportion seems to be 3:1. Importantly, fats in the KD should be well balanced in terms of saturated, mono- and polyunsaturated fatty acids, while proteins are kept to the minimum requirements for growth. The higher the proportion, the more restrictive and theoretically more effective the diet is. Furthermore, the eating plan drastically reduces the intake of processed, chemically treated foods and focuses on fresh, nutrient-dense meals including meat, fish, vegetables, and healthy oils. Protein from lean meat, fish, poultry, and cold cuts is consumed in moderation. The low-carbohydrate vegetables and fruits include spinach, broccoli, cauliflowers, cabbage, cucumbers, peppers, tomatoes, leeks, radishes, green lettuce, grapefruits, apples, tangerines, oranges, strawberries, and avocados. Olive oil, butter, mayonnaise, as well as fatty meat and dairy are the main sources of lipids. Because such a diet is deficient in some nutrients, patients should be provided supplements of calcium, vitamin D, iron, folic acid, zinc, selenium, and copper [2,3,8,14].

The MAD and LGIT were developed as less restrictive and more palatable options to the classic KD. They are usually recommended in children to increase their compliance to the therapy. With a 1:1 or 2:1 ketogenic ratio, an MAD provides patients with much better physical development. A diet with such a ratio delivers unlimited protein, fat, and calorie intake, while the daily carbohydrate restriction is 10–20 g in children and 15–25 g in adolescents. An LGIT, without any fixed ketogenic ratios, restricts daily carbohydrate intake to 40 to 60 g using products with a lower-than-50 glycemic index. This diet was used for the first time in 2005 for two adolescent boys, who responded well to the cKD, but could not continue this treatment because of unacceptable adverse effects. Importantly, neither an MAD nor an LGIT require an initial fasting phase or hospitalization [3,6,15,16].

**Figure 1:**
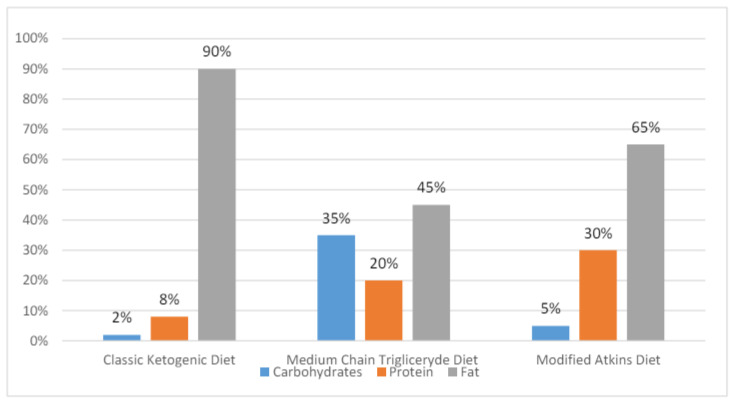
Percentage compositions of the classic KD and its modifications [2,3,6,8,14,15,16].

The MCT diet is an enhanced variant of the classic KD with 45–60% of dietary energy coming from fatty acids. The ketogenicity of the MCT is greater than that of classic KD, as medium-chain fatty acids are easily transported into the cells and rapidly metabolized. As its is more ketogenic, the MCT allows for a lower fat content and an increased daily intake of proteins and carbohydrates, which in turn raises diet tolerance and reduces gastrointestinal adverse effects. Medium-chain triglycerides are produced by the hydrolysis of coconut and palm oils, with their representatives being decanoic (capric) acid and octanoic acid. These fatty acids are directly absorbed through the intestinal wall and transported to the liver. β-Oxidation quickly breaks them down forming β-hydroxybutyrate, acetoacetate, and acetone as the three primary ketone bodies [17,18]. The percentage compositions of the KDs are presented in Figure 1.

The dietary protocol most prescribed to children is classic KD (60%). It is followed by the MAD (25%), primarily used in adolescents and adults, and the MCT (10%), indicated in patients with compliance problems. The LGIT is prescribed in around 5% of epilepsy patients. All KDs are being explored more and more as possible treatments for a variety of conditions other than drug-resistant epilepsy, like Parkinson’s disease, autism spectrum disorders, endocrine disorders, Alzheimer’s disease, malignant glioma, migraine headaches, and motor neuron disease. However, a recent meta-analysis did not confirm benefits from LGIT in adjuvant cancer therapy [14,19].

Despite the availability of different KD variants, the attrition rate still remains a serious problem. The main reasons of withdrawal are adverse effects and a lack of effectiveness. Not surprisingly, retention is higher in infants due to the easier control of the diet by their caregivers. Patients with drug-resistant seizures need long-term dietary therapy continued for approximately 2 years. In general, 1 and 3-month therapy is considered as a short-term option, while 6, 12, and 24-month therapy is considered as a long-term treatment. Responders are defined as patients who achieved a ≥50% reduction in seizure frequency [20,21].

## 2. Mechanisms of Action of the KD

The fundamentals of the KD’s anticonvulsant effects still remain unclear, although many potential mechanisms have been discovered. In the past, the direct effects of ketone bodies were taken into consideration; however, the poor correlation between the plasma concentrations of ketone bodies and antiseizure effects is presently emphasized. Other potential mechanisms include 1. the direct antagonistic action of decanoic acid on AMPA glutamatergic receptors, 2. the increased levels of neurotransmitters (norepinephrine, dopamine, serotonin, galanin, and neuropeptide Y) involved in the antiseizure effect, 3. decreased glutamatergic synaptic transmission, 4. the ATP-sensitive potassium channel opening, 5. the improvement of mitochondrial function, 6. histone hyperacetylation, and 7. the suppression of the mammalian target of rapamycin (mTOR) pathway. Less frequently assumed factors comprise 1. the activation of voltage-gated K channels by polyunsaturated fatty acids, 2. an increased production of the brain-derived neurotrophic factor (BDNF) linked to AMP kinase (AMPK) and mTOR signaling, and 3. the remodeling of the gut microbiome increasing the GABA/glutamate ratio in the hippocampus [6,16,22,23,24].

**Figure 2:**
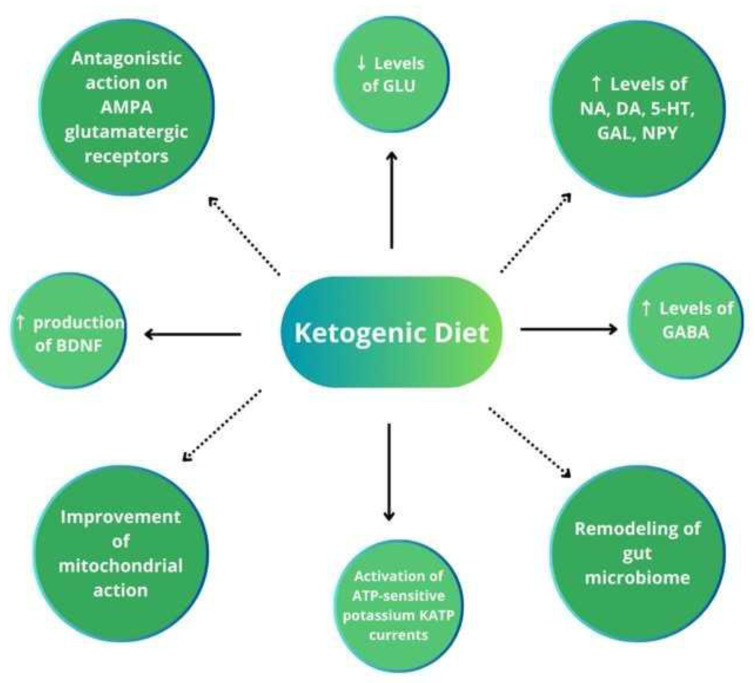
Leading mechanisms of action of the ketogenic diet leading to neuronal hyperpolarization [22,23,24,25]. ↑, increased; ↓, decreased; AMPA, α-amino-3-hydroxy-5-methyl-4-isoxazolepropionic acid; GLU, glutamate; NA, norepinephrine; DA, dopamine; 5-HT, serotonin; GAL, galanin; NPY, neuropeptide Y; BDNF, brain-derived neurotrophic factor; GABA, γ-aminobutyric acid; ATP, adenosine triphosphate.

Several reports indicate that the KD maintains a balance between the brain’s excitatory and inhibitory neurotransmitter systems, increases GABA concentrations in the brain, and induces neuronal hyperpolarization, e.g., by activating potassium channels. Ketone bodies may induce cataplerosis (the removal of intermediate metabolites) in the citric acid cycle, increase mitochondrial oxidative phosphorylation and ATP production, as well as enhance the synthesis of antioxidants. Reduced glucose levels in patients on the KD decrease cellular pyruvate/oxaloacetate concentrations, which in turn can hyperpolarize neurons through the activation of neuronal ATP-sensitive potassium K_ATP_ currents. The K_ATP_ channels play the role of a sensor for the state of cellular energy. In the feedback mechanism, the channels reduce neuronal firing when energy levels are low. K_ATP_ currents are regulated by the BCL2-associated agonist of cell death (BAD), a protein involved in the control of apoptotic processes and glucose metabolism. The activation of the BAD decreases glucose metabolism and activates K_ATP_ channels, thus contributing to the reduction of excessive neuronal firing, protection from the onset of spontaneous recurrent seizures, and enhanced neuroprotection. In animal models, glucose analogue 2-deoxy-D-glucose proved to be a unique anticonvulsant action mediated through the partial inhibition of glycolysis [23,25]. For a review, see Figure 2.

When it comes to the direct anticonvulsant effect of ketones, acetone caused neuronal hyperpolarization and reduced neuronal excitability by activating double-pore ion channels for potassium ions (K2P channels), which are crucial for preserving the resting membrane potential and controlling the excitability of the nerve cell membrane. Ketone bodies were also reported to decrease the release of some neurotransmitters, such as glutamate, norepinephrine, or adenosine. Another theory assumes that ketone bodies and free fatty acids serve as epigenetic factors regulating the expression of seizure-related genes and reducing DNA methylation through increasing levels of adenosine. Interestingly, in amoeba Dictyostelium, medium-chain-fatty acids regulated phosphoinositide signaling analogously but more potently than valproate [3,17,20,23].

Furthermore, the neuroprotective action of the KD can be due to raised calbindin levels, the buffering of intracellular calcium, and the suppression of apoptotic factors like caspase 3. This diet treatment increases the removal of free radicals, thus reducing oxidative stress-induced damage. This might be at least partially achieved by the raised glutathione levels and enhanced mitochondrial antioxidant capability. Furthermore, the KD suppresses the mTOR and the nuclear factor erythroid 2-related factor 2 (Nrf2) in both pathways reducing oxidative stress [19,23,24,26].

The anticonvulsant action of the MCT diet appears to be dependent on the effects of decanoic acid, but not those of octanoic acid. Numerous in vivo studies demonstrated that decanoic acid, applied at clinically relevant concentrations, exhibited antiepileptic properties by reducing excitatory postsynaptic currents. In two ex vivo rat models of epilepsy, decanoic acid, but not β-hydroxybutyrate or acetone, provided antiseizure activity through direct non-competitive antagonistic action on glutamatergic AMPA receptors, preferably their Glu2/3 subunits. Decanoic acid was also reported to activate the nuclear receptor PPARg leading to the mitochondrial proliferation and activation of mitochondrial complex I. In experimental conditions, decanoic acid, but not octanoic acid, enhanced the antiseizure action of valproate. On the other hand, the simultaneous administration of decanoic and octanoic acids resulted in a synergistic anticonvulsant interaction, which may contribute to the better therapeutic effect of the MCT diet [17,22,27,28,29].

## 3. The Effectiveness of the Ketogenic Diet

As mentioned, much more attention is paid to intractable seizures in infants, children, and adolescents when compared to adults. However, regardless of the age of the patients, the available meta-analysis emphasizes the paucity of randomized clinical trials, low-quality methodologies, low group sizes, and too much diversity in participants (with regard to age, seizure type, and previous treatment). All these factors make statistical analysis and correct concluding difficult.

One randomized clinical trial revealed MAD efficiency in adults with refractory focal epilepsy. The authors found a moderate difference between the diet and control groups only if the parameter of a >25% seizure frequency reduction was taken into account. However, seizure response was considerably variable, probably due to fluctuating decreases in the serum concentrations of discontinued antiepileptic drugs. The same investigators evaluated the quality of life of their patients after a 12-week diet intervention and noted significant improvement in the mean total score, which was not related to the diet-induced weight loss. Such a result was somewhat surprising, as the diet treatment is demanding, time-consuming, more expensive, and deprives the patient of their favorite meals. Probably, the diet, in contrast to oral drugs, could give the patients a sense of control over their own health. Additionally, KDs have the potential to treat adult status epilepticus. A systematic review addressed the effectiveness of KD therapy in adult patients with epilepsy. In a large prospective multicenter study, out of 15 adult patients with super resistant status epilepticus, treated with a KD at a 4:1 ratio for 10 days, 11 participants presented a resolution of status epilepticus in less than 5 days. In a case series of 10 adults with super resistant status epilepticus, treated with KDs at 3:1 or 4:1 proportions, 9 patients achieved the cessation of seizures at a median of 3 days. In another study, 11 patients with the same condition were treated with KDs and all of them experienced the cessation of status epilepticus. In general, the analyzed observational studies revealed that 31 of 38 (82%) adult patients with resistant or super resistant status epilepticus accomplished the cessation of seizures during KD therapy. The effectiveness of such treatment was similar to that observed in pediatric patients (75%). Data from 13 case reports and series showed that the KD successfully ceased the occurrence of seizures in adults with resistant or super resistant status epilepticus. The time to status epilepticus resolution ranged from 4 to 25 days. In another review, a few randomized control trials and several recent observational trials were described. The presented studies assessed the effectiveness of different variants of a KD (the classic KD, an MCT, an MAD, and an LGIT) in the treatment of adult patients with drug-resistant epilepsies. The first one assessed the efficacy of 2-month adjunctive therapy with an MAD in 34 patients with focal and generalized seizures. The response rate was 35.5%, but none of the patients achieved seizure freedom.

In another randomized control trial, 37 patients with resistant focal and multifocal epilepsy were randomized to the MAD group. Four participants dropped out due to an increased severity of seizures or non-compliance. The remaining patients experienced a significant but moderate 25–50% reduction in seizure frequency. A further study evaluated the influence of a ketogenic formula (KetoCal^®^) on seizure control in 80 adults with refractory focal or generalized convulsions. After 1 month, 30 patients (37.5%) achieved a ≥50% seizure reduction, compared to 23 patients (29%) at 2 months and 20 patients (25%) at 6 months. The ketogenic formula did not affect the responder rate, however, it improved the patient compliance. A prospective observational study evaluated the responder rate to the long-term modified KD in 40 adults with drug-resistant focal and generalized epilepsies. The diet effectiveness was assessed every 3 months up to 12 months. Resultantly, five patients (12.5%) achieved a period of freedom from all types of seizures that was three times longer than that before the diet, whereas fifteen patients (38%) experienced a ≥50% seizure frequency reduction. However, retention rates decreased with time from 60% at 3 months to 43% at 6 months and only 29% at 12 months. Additionally, two meta-analyses of data on adult patients treated with various types of KDs were published. The first one, based on the analysis of 16 trials including 338 subjects, demonstrated a 13% combined rate of seizure freedom and a 53% responder rate. Patients were treated with the KD, MAD or MAD/KD. Another meta-analysis evaluated 12 studies in which the overall responder rate ranged from 13 to 70%. The respective combined rates for the classic KD and the MAD were assessed as 52% and 34%. Other benefits of the KD, apart from the reduction of seizure frequency, were a decrease in seizure severity as well as an improvement in mood, drive, and quality of life [10,19,30,31,32].

Among children with drug-resistant epilepsy, roughly 8.4% from the group on the KD experienced a seizure free status, while a ≥50% seizure frequency reduction was achieved in 61.4–72% of patients [33,34]. In West syndrome (infantile spasms), encouraging, though limited data showed a ≥50% reduction in seizure frequency in 48.31% of the KD group’s participants compared to 15.81% of those in the control group. After a 36-month follow up, a ≥50% seizure frequency reduction was observed in 63% of KD-treated children. Better seizure control in infants vs. other age groups is thought to be related to more suitable diet compliance being strictly controlled by caregivers. Furthermore, the time trend analysis showed that the number of pediatric patients achieving ≥50 and ≥90% seizure frequency reductions gradually decreased in favor of an increase in the number of patients achieving a seizure free status. The treatment of infantile spasms with the classic KD appeared to be as similarly effective as ACTH therapy—62% of patients in the classic KD group vs. 69% in the ACTH group achieved ≥90% seizure control within 28 days. The concomitant treatment with the MAD and the ongoing anticonvulsant therapy appeared to be superior to the antiepileptic treatment alone. Three months after diet initiation, 83.3% of patients achieved a ≥50% seizure frequency reduction, while 33.3% experienced a seizure-free state [35]. The same group of researchers conducted an open labeled randomized clinical trial, where they compared the efficacy and tolerability of the MAD vs. the classic KD in children up to 3 years of age suffering from West syndrome resistant to steroid therapy and a variety of antiepileptic medications, including vigabatrin, topiramate, zonisamide, and benzodiazepines. The effects of the two diets were comparable at 4 and 12 weeks. However, there was a tendency for the MAD to be more efficacious at 4 weeks, while the classic KD tended to be at 12 weeks. However, small group sizes were the most important limitation of this study [36].

Earlier comparative randomized clinical trials reported that both the classic KD and MCT were similarly effective in the treatment of pediatric refractory epilepsy. However, according to the authors, for patients younger than two years old, the classic KD seems to be a better option, as the hyperexcitable brains of infants require fast intervention to avoid the development of epileptic encephalopathy [37]. A recent randomized clinical trial showed that both the classic KD and the MAD were almost equally effective in seizure control. In detail, 60% in the classic KD group and 53.33% in the MAD group achieved a seizure-free state, while the remaining patients experienced a ≥50% seizure frequency reduction. Adverse effects were mild and manageable, with lipid profiles maintaining an acceptable range in both groups. Patients had improved growth parameters and EEG patterns. Interestingly, the introduction of the classic KD improved seizure outcomes in one-third of pediatric patients, in whom the MAD therapy appeared to be inadequate. Finally, several meta-analyses showed that both the classic KD and the MAD were effective in achieving three main parameters: a ≥50% seizure frequency reduction, a ≥90% seizure frequency reduction, and a seizure-free state in patients with West syndrome at 3 and 6 months after diet initiation. However, the MAD appeared to be better tolerated, providing a higher probability for a ≥50% seizure frequency reduction and a similar probability for a ≥90% seizure frequency reduction. Therefore, it may be a more advantageous therapeutic option than the classic KD [38,39].

Another randomized clinical trial showed that achieving a seizure-free status at 12 weeks was comparable between the MAD and the LGIT diet used in the treatment of pediatric intractable epilepsy. At the same time, the number of patients with a ≥50% seizure frequency reduction was significantly higher in the LGIT group compared to the MAD-treated individuals (73.3% vs. 43.3%) [40]. Also, in children with drug-resistant epilepsy, the classic KD, MAD, and LGIT appeared to be more effective than care as usual with regard to a ≥50% seizure frequency reduction. However, the classic KD and the MAD were the most likely to achieve a ≥90% seizure frequency reduction and seizure freedom in the short term. The comparison of different variants of KDs and different ketogenic ratios was unreliable because of the lack of good quality results. Very few clinical studies were conducted with patients on the LGIT diet. Similarly, a very small number of studies were focused on the long-term outcomes of the KD, MAD, or LGIT. There are no data comparing different KD initiation protocols, with the exception of one study favoring a gradual vs. a fasting initiation. In children with resistant seizures, a gradual initiation provided an efficacy comparable to fasting initiation and decreased the rate of adverse effects [41].

There are also reports on the advantageous action of the KD in patients with Dravet syndrome and Lennox–Gastaut syndrome. In Dravet syndrome, children who had not responded to several anticonvulsant drugs, including stiripentol, were subjected to the KD. In the first month, a ≥50% seizure frequency reduction was achieved in around 67% of patients. Moreover, in approximately 53% of patients, the effectiveness lasted for up to six months. In contrast, the KD was not effective in children with febrile infection-related epilepsy syndrome (FIRES), a catastrophic epileptic encephalopathy with an as-of-yet-undefined etiology [7,42]. A meta-analysis of results in patients with Dravet syndrome showed that the KD was effective with the pooled efficacy rate for a ≥50% seizure frequency reduction of 63% at 3 months, 60% at 6 months, and 47% at 2 months, respectively. The efficacy of the KD decreased with the prolongation of treatment time, probably due to the loss of compliance. The occurrence of long-term adverse effects was greater than that of short-term undesired events. The antiepileptic effects of the KD turned out not to be lower than those of antiseizure drugs, if both options were compared as a first-line therapy. Furthermore, the KD was proven to reduce further polytherapy with antiseizure medications [41]. Also, a considerable proportion of patients with Lennox–Gastaut syndrome experienced a ≥50% seizure frequency reduction and a seizure-free state when following any type of KD [24]. One retrospective single-arm meta-analysis showed the effectiveness of the KD in children with cyclin-dependent kinase-like 5 (CDKL5)-related epilepsy, with the responder rate of 18%. Nevertheless, all included studies were small-sized and of rather low quality [43].

Interestingly, children with intractable epilepsy who were on the classic KD often experienced occasional breakthrough seizures after 1 month of seizure freedom. This quite common phenomenon occurred in 82% of initially seizure-free patients, with only a 3% likelihood of remaining completely seizure free at 18 months. However, the continuation of the diet stopped the seizures. Therefore, it is recommended to continue the KD in such patients even after the subsequent seizure recurrence. The timing of the discontinuation of antiepileptic drugs (early or late) did not affect the reappearance of seizures. An important argument for early treatment with the KD in children, particularly in those with Dravet syndrome, is the improvement of cognitive parameters (mostly alertness) and behavioral disorders. A randomized clinical trial demonstrated that patients on the KD were more active, more productive, and less anxious at the 40-month follow-up. Another clinical trial reported higher productivity, reduced hostility, and better cognitive functioning. However, these positive effects may result from not only the diet itself, but also from the reduction of seizures [6,16,20,44].

Despite such very promising results, the final assumptions should be much more balanced. A detailed meta-analysis of randomized clinical trials emphasized the lack of blinding and various risks of bias in these studies. The authors showed that there were no reports of seizure freedom in adults following KDs, however, they may be up to five times more likely to experience a ≥50% seizure frequency reduction compared to adults treated with antiepileptic drugs. Children on KDs can be up to 3 times more likely to achieve seizure freedom and up to 6 times more likely to experience a ≥50% seizure frequency reduction compared to children subjected to usual care. Although, in one study, over 50% of the pediatric patients on the classic KD became seizure free, this rate dropped to only 15% when they were treated with a less-restrictive MAD. In another study, 85% of children on the classic KD (4:1) had a significant ≥50% seizure frequency reduction at 3 months compared to only around 50% of patients on the MAD. Reported rates of seizure freedom at 3 months reached 55% in a 4:1 KD group. Studies evaluating the efficacy of the MAD in children reported seizure freedom rates up to 25% and ≥50% seizure freedom reduction rates of up to 60%. In adults, the effect of the MAD is less clear, with no participants experiencing seizure freedom and controversial results in ≥50% seizure frequency reductions (rates from 35% to 8%). Although there was some evidence of greater antiepileptic efficacy for 4:1 KD groups over lower ratios, the highest ketogenic ratio was associated with the higher rate of adverse effects. Summing up, the collective evidence suggests an advantageous effect for the use of KDs for the treatment of drug-resistant epilepsies in children. However, the limited number of studies in all age groups, especially in adults, the small sample sizes, and the generally short-term follow-up resulted in a low to very low certainty of evidence for the majority of outcomes. Randomized controlled trials comparing all KD variants reported adverse effects of varying frequencies and intensities, from short-term gastrointestinal disturbances to different long-term complications. Patients usually complained of vomiting, constipation, diarrhea, and increased cholesterol levels. The adverse effects associated with the MAD may initially appear to be less than those related to the classic KD, but further studies are required to confirm this statement. Attrition rates remained a problem in all types of KDs, the reasons for which are mainly the lack of treatment efficacy and the intolerance of the diet. Although one study found no significant difference in seizure frequency rates between gradual-onset and fasting-onset KDs, further large-scale studies are required to verify it. The effect of KDs on quality of life, cognition, and behavior also requires further investigation. Because of the lack of reliable evidence for the use of KDs in adults or infants with epilepsy, additional research is needed in this area. Also, the assumption that more palatable diets, such as the MAD, may be similarly as effective as the classic KD requires confirmation [45].

The further systematic review and meta-analysis of the results obtained from patients with intractable seizures on the MAD revealed that children and adults were about 6 and 4 times, respectively, more likely to achieve a ≥50% seizure frequency reduction, and 5 times more likely to achieve a seizure-free state when compared to being on a regular diet. Patients developed no essential adverse effects. These more advantageous results can be explained by the larger sample sizes of the analyzed trials. The most common adverse effects experienced by patients were constipation, diarrhea, and lethargy [46].

The classic ketogenic diet versus further antiseizure treatment of infants with drug-resistant epilepsy (KIWE) study group conducted a multicenter, open-labeled, randomized clinical trial that compared the classic KD vs. further anticonvulsant medication in children up to 2 years of age. Investigators used classic KD protocols from 4:1 to 2:1 ratios and gradual diet initiation. Resultantly, the classic KD did not differ in efficacy and tolerability to further pharmacological treatment, and it appeared to be well tolerated in the observed patients. According to the authors, the classic KD could be a treatment option in infants whose seizures were not controlled by two antiseizure medications [47].

The vast majority of the literature concerns the short-term (up to 3 months) use of a ketogenic diet. Long-term treatment with a KD was described in only a few publications. Interestingly, 8-month- vs. 24-month KD therapy was compared in infants with West syndrome who positively responded to the diet. As a result, the short use of the KD gave outcomes and recurrence rates comparable to those achieved in the long-term therapy. Not surprisingly, fewer growth disorders and chronic adverse effects were observed in the 8-month group [19]. The total retention rates usually decrease in time, like in the study on childhood epilepsy in which this parameter dropped from 45.7% after one year of therapy to 29.2% after 2 years. The main reason for the diet’s discontinuation was the lack of efficacy [48]. Similarly, the retention rate in adults on the KD therapy was reduced from 79.0% at 6 months to 57.9% at 12 months after diet initiation. These results were not significantly different from the retention rates for children (82.8% at 6 months and 68.9% at 12 months) [30]. Decreasing adherence is probably one of reasons for the small number of publications on the long-term use of KD. Murphy [31] reported the effects of the KD administered for 1 to 3 years. In the analyzed studies, the diet was extremely effective in controlling seizures and allowed for a significant reduction in the doses of antiepileptic drugs or their complete discontinuation. Importantly, seizure frequency and severity may be reduced or even abolished for a long time after stopping treatment. The adverse effects of the KD reported in this study were mild to moderate and controllable. An interesting and unique retrospective review of 28 children treated with the KD for more than 6 years was done in Johns Hopkins Hospital. After such a long-term therapy, 24 children experienced a ≥90% seizure frequency reduction. Also, the overall tolerability was maintained after the prolonged use of the KD. Ten pediatric patients improved their height from the 10th to the 23rd centile (*p* = 0.001). Kidney stones developed in 7 children, while skeletal fractures occurred in 6. In the lipid profile, the mean total cholesterol and low-density lipoprotein concentrations were moderately increased (201 mg/dL and 129 mg/kg, respectively), while high-density lipoprotein (54 mg/dL) and triglyceride (97 mg/dL) levels were normal [31,32]. A brief comparison of clinical trials using KD is provided in the Table 2.

## 4. Adverse Effects

According to some authors, the KD-evoked side effects can be even milder than those brought on by some antiepileptic medications. Nevertheless, approximately 10% of patients discontinue the classic KD because of its unpalatability and adverse gastrointestinal effects, the most common being diarrhea, constipation, nausea, and vomiting. Long-term adverse effects include dehydration (46.5%), gastrointestinal issues (38.8%), renal stone formation (3.1%), and liver damage (2.3%). A smaller proportion of patients experience far more severe adverse effects, such as electrolyte imbalances, hypoglycemia, hypoproteinemia with edema, kidney dysfunction, severe liver damage (mostly due to interactions with antiepileptic drugs), and cardiomyopathy. Regarding the hepatotoxicity, the elevation in liver enzymes becomes apparent with the diet’s initiation, hepatic steatosis develops most often within 6 months, while gallstone formation is diagnosed usually after 12 months of therapy. Biochemical disturbances, like hypocalcemia, hyperuricemia, hypocitruria, and metabolic acidosis (appearing during diet initiation) may predispose a patient to kidney stone formation. In children, the classic KD can be a reason of growth retardation. In turn, adolescents can experience disorders of bone mineralization sometimes leading to osteomalacia (14.7%). With raised cholesterol and triglyceride plasma concentrations, this diet appears to raise the risk of atherosclerosis development. Nevertheless, most adverse effects caused by the classic KD treatment have been reported to be transient, or at least easy to prevent and treat. It is worth noting that critically ill adults with status epilepticus tolerated the KD therapy well. Reported adverse effects included gastrointestinal symptoms, metabolic acidosis, hyperlipidemia, hypoglycemia, nephrolithiasis, and a greater propensity to infections [2,3,9,10,31,32,54,55,56].

In contrast to the classic KD, the MAD, MCT, and LGIT are evaluated as being more palatable and tolerable. On these diets, patients can eat more varied food and have less undesired gastrointestinal or growth disturbances. Moreover, diets other than classic KD do not require hospital conditions during their initiation. Large supplementations of micro- and macroelements, as well as caloric, protein, or fluid restrictions are also unnecessary. Patients on the MAD experienced mild adverse effects including constipation, diarrhea, vomiting, bloating, abdominal pain, and anorexia. One patient was reported to develop osteopenia. Additionally, only a few serious consequences from the MAD were observed. The adverse reactions were mostly temporary, well managed by cautious care, and did not require that the patient stop the diet. Common negative effects included constipation, vomiting, anorexia, weight loss, and raised triglycerides and cholesterol. However, patients have significantly lower total cholesterol/high density lipoprotein ratios compared to those on the cKD. Patients treated with the MCT diet are more likely to experience abdominal bloating and diarrhea, while patients on the classic KD are more prone to constipation. One case study revealed that an *iv* MCT diet led to liver dysfunction, severe iron deficiency, and transient triglyceride and cholesterol increases. The most common undesired effect of the LGIT is temporary diarrhea. Among laboratory abnormalities, hypercholesterolemia, increased alanine aminotransferase, lipase, and blood urea nitrogen were most commonly observed. These conditions did not, however, require additional management or medications [2,3,24,57,58]. Partial dehydration, caused by the natriuretic effects of the KD, and a hypoproteinemia-evoked decrease in plasma protein binding can elevate the plasma and brain levels of antiepileptic drugs, enhancing their therapeutic, but also their toxic effects. Therefore, the possible interactions between the KD and AEDs should be correctly assessed, especially in that most patients continue pharmacological treatment alongside the diet therapy. The carbonic anhydrase inhibitors (e.g., topiramate and zonisamide) were reported to enhance hypercalciuria, hypocitraturia, urine acidosis, and the risk of urolithiasis. To avoid renal dysfunctions, patients should be closely monitored, rehydrated, and supplemented with potassium citrate. In a large retrospective study, the 3-month ketogenic regime in patients treated with antiepileptic medications (e.g., lamotrigine) decreased the effectiveness of the diet. The conclusion was that anticonvulsants should be discontinued after at least 4–8 weeks after diet initiation. Also, steroid therapy, including the use of adrenocorticotropic hormone (ACTH), was reported to reduce dietary effects, but this observation was not confirmed in more recent studies [3,8,58,59].

## 5. Discussion

The KD appears to be increasingly established in the treatment of drug-resistant epilepsy, gaining popularity among dietitians, neurologists, and patients. It encourages scientists to find out more about its mechanisms of action, efficacy, and tolerability. Pregnant women with epilepsy or those planning to become pregnant, as well as parents of children with epilepsy, are particularly interested in this form of treatment, given the lack of teratogenicity and undesired effects related to pharmacological therapy. For ethical reasons, the KD is always initiated as an add-on therapy. During treatment, antiepileptic drugs may be applied at lower doses or even withdrawn. The KD and its variants turns out to be successful in the treatment of intractable epilepsy syndromes in children and adolescents, including West syndrome, myoclonic-astatic epilepsy, Dravet syndrome, tuberous sclerosis, Landau–Kleffner syndrome, Rett syndrome, and Lennox–Gastaut syndrome. Although the diet was effective in treating generalized epilepsy, much less efficacy was observed in complex partial seizures. In general, 8–10% of children achieved seizure freedom, while 61–72% gained a ≥50% seizure frequency reduction. Only a few studies referred to the use of the KD in adults. Primary indications include resistant and super resistant status epilepticus as well as focal, multifocal, and generalized epilepsy. In contrast, all variants of KDs are completely contraindicated in the case of errors related to lipid and pyruvate metabolisms. Similarly, only a few publications reported the long-term efficacy and tolerance of the KD in patients who were treated for 1 to 6 years. Seizure frequency and severity were reported to be much more reduced (a ≥50% seizure frequency reduction in 61–72% of patients) or even abolished (in 12–13%). Patients experienced mild adverse effects and had moderate hypercholesterolemia. Such scarce data on the long-term effects of the KD are primarily due to low adherence rates. The diet is assessed by patients as being unpalatable and difficult to follow. Nevertheless, its efficacy in intractable epilepsy is often considered to be worth the effort, especially in that for some individuals, the diet seems to be the only effective treatment. In some cases it is possible to switch from the unpalatable KD to the more palatable MAD. Another reason for low adherence is the occurrence of adverse effects. Nevertheless, the negative consequences of the diet are generally minor and controllable. Importantly, seizure activity may be reduced or eliminated for long periods after the diet is stopped. Supporters of treating epilepsy with diet changes claim that the short- and medium-term advantages of the KD on seizures are equivalent to those of contemporary antiepileptic therapy. Additionally, improvements in mood, behavior, and cognition unrelated to seizure control have been reported by patients on the diet. Cognitive improvement was observed in the areas of alertness, attention, and global cognition [12,30,31,32,60].

The KD detractors point out that no randomized, double-blind, placebo-controlled clinical trial has yet demonstrated the diet’s efficacy. Moreover, the quality of the evidence in available studies of either adults or children was very low. However, available, randomized, open-label clinical trials have yielded some promising outcomes. All variants of the KD, including the classic KD, the MAD, and the LGIT, have a proven effectiveness for ≥50% seizure frequency reduction rates compared to care as usual. The classic KD and MAD were more effective for achieving a short-term ≥90% seizure frequency reduction and seizure freedom than LGIT. According to some meta-analyses, the MAD, as it is better tolerated, provides a higher probability for a ≥50% seizure frequency reduction, and a comparable probability for ≥90% seizure frequency reduction and seizure freedom rates. It appears to be a better option than the classic KD in infants, children, and adolescents with intractable epilepsy. Nevertheless, the evidence for the effectiveness of the KD in epilepsy patients still remains poor, mainly because of the lack of double-blind trials and the too small group sizes. There is also a paucity of trials focused on the intermediate and long-term outcomes of KD therapy [2,3,14,16,20,26,46,47,55].

Further randomized clinical studies are required to confirm the cognitive and developmental effect of KDs. Better adherence to methodological standards is necessary for a reliable meta-analysis of the data obtained. It would be advantageous to estimate correlations between seizure frequency reduction and concentrations of ketone bodies. The effects of lowered glucose on adequate gene and protein expression seem to be further areas of interest. Additional findings are necessary to confirm whether more palatable variants of the KD can manage seizures to a satisfactory extent. To guarantee appropriate therapeutic effects and prevent patients from entering ketosis and having seizures, daily collaboration between a neurology service, a critical care team, and a nutritionist is necessary.

## Figures and Tables

**Table 1:** Recommendations for the ketogenic diet in children (adapted from [5]).

Conditions for which the KD Offers ≥70% SFR	Conditions for which the KD Offers ≥70% SFR
Angelman syndrome	Adenylosuccinate lyase deficiency
Complex 1 mitochondrial disorders	cyclin-dependent kinase-like 5 (CDKL5) encephalopathy
Dravet syndrome	Childhood absence epilepsy
Doose syndrome	Cortical malformations
Glucose transporter protein 1 (GLUT 1) deficiency syndrome	Epilepsy of infancy with migrating focal seizures
Febrile infection–related epilepsy syndrome	Epileptic encephalopathy with continuous spike-and-wave during sleep
Ohtahara syndrome	Glycogenosis type V
Infantile spasms	Juvenile myoclonic epilepsy
Formula-fed (solely) children or infants	Lafora body disease
Pyruvate dehydrogenase deficiency (PDHD)	Landau-Kleffner syndrome
Super-refractory status epilepticus	Lennox-Gastaut syndrome
Tuberous sclerosis complex	Phosphofructokinase deficiency
	Rett syndrome
	Subacute sclerosing panencephalitis

**Table 2:** A comparison of the research on the ketogenic diet and its varieties (2008–2024).

Authors	Clinical Trial	Characteristic	Results
Manral et al., 2023 [15]	The research compared the effectiveness of standard antiepileptic therapy with its combination with the MAD.	prospective, controlled, randomized trial	The MAD/standard therapy group showed significant improvement in all seizure and behavioral parameters compared to the control group.
Neal et al., 2009 [37]	The goal of this research was to evaluate the efficacy of the KD.	prospective, randomized, non-blinded, controlled trial	The KD effectiveness in the treatment of intractable seizures in children has been proven. However, all benefits and negatives should be considered individually.
Kim et al., 2016 [38]	The research evaluated the effectiveness, safety, and tolerability of the MAD and the classic KD in the treatment of pediatric intractable epilepsy.	randomized clinical trial	The MAD is recommended to children with persistent epilepsy, while the classic KD seems to be better for patients under two years as the initial dietary therapy.
Titre-Johnson et al., 2017 [49]	The study explores the use of the KD for treating epilepsy in children less than two years of age.	randomized trial	When several antiepileptic drugs have failed to control an infant’s seizures, the adjunctive treatment with the KD is more successful than drugs alone.
McDonald et al., 2018 [50]	The research investigated whether adding a ketogenic formula to the MAD during the first month of treatment improves compliance and reduces seizures more than the MAD alone.	randomized trial	The introduction of a ketogenic formula in the initial month of the MAD regimen enhances the likelihood of long-term MAD adherence compared to the MAD alone.
Cervenka et al., 2017 [51]	The analysis evaluated the safety, effectiveness, and feasibility of the KD in the treatment of individuals with super refractory status epilepticus.	prospective, multicenter research in adult patients with epilepsy	Super refractory status epilepticus stopped in 73% of patients, usually within a week of KD initiation.
Taub et al., 2014 [52]	The study evaluated the clinical characteristics of children on the KD who experienced at least one month of seizure freedom.	controlled trial	This study offers proof in favor of keeping patients who achieved a seizure-free state on the KD even when they develop breakthrough seizures. The retrospective design of the study limits the evaluation of possible seizure recurrence.
Raju et al., 2011 [53]	The purpose of this study was to evaluate the safety and efficacy of the diet using different lipid to non-lipid ratios to treat childhood-resistant epilepsy in patients with coronary heart disease.	randomized, non-blinded, open-label, parallel, controlled trial	Controlling seizures may also benefit from a ketogenic ratio of less than 4:1.

## Data Availability

The data presented in this study are available on request from the corresponding author. The data are not publicly available due to privacy.
